# Supplementary material for: The Distribution of Neospora caninum Secretory Proteins in Mouse and Calf Brains
Source: Microorganisms. 2025 Aug 22;13(9):1970. doi: 10.3390/microorganisms13091970 (PMC12471860; doi:10.3390/microorganisms13091970)
Supplement: Supplementary file 1 [file microorganisms-13-01970-s001.zip › microorganisms-3777056-supplementary.pdf]

Supplementary materials

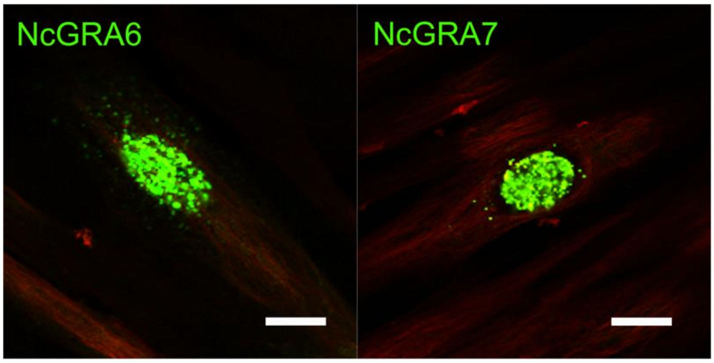

Figure S1. Confocal microscopy images of NcGRA6 and NcGRA7. NcGRA6 and NcGRA7 (green signals) are observed in the cytoplasm of infected cells. Red: anti-β tubulin. Bar= 20 μm.

Table S1. Quantification of immunostaining patterns in parasitophorous vacuoles across individual animals.

Animal	ID	Antibody	TypeA	TypeB	Total PVs*
Mouse	1	NcSAG1	45	0	45
	2	NcSAG1	77	0	77
	3	NcSAG1	57	0	57
	4	NcSAG1	100	0	100
	5	NcSAG1	40	1	41
	1	NcCYP	48	2	50
	2	NcCYP	46	2	48
	3	NcCYP	47	1	48
	4	NcCYP	71	0	71
	5	NcCYP	43	0	43
	1	NcPF	35	0	35
	2	NcPF	43	0	43
	3	NcPF	62	0	62
	4	NcPF	97	3	100
	5	NcPF	44	2	46
	1	NcGRA6	7	30	37
	2	NcGRA6	7	40	47
	3	NcGRA6	4	26	30
	4	NcGRA6	4	96	100
	5	NcGRA6	4	48	52
	1	NcGRA7	1	30	31
	2	NcGRA7	1	61	62
	3	NcGRA7	1	39	40
	4	NcGRA7	0	100	100
	5	NcGRA7	0	46	46
Calf	1	NcSAG1	13	0	13
	2	NcSAG1	25	0	25
	1	NcCYP	14	0	14
	2	NcCYP	30	0	30
	1	NcPF	11	0	11
	2	NcPF	26	0	26
	1	NcGRA6	2	10	12
	2	NcGRA6	1	42	43
	1	NcGRA7	0	10	10
	2	NcGRA7	1	22	23

*PVs, parasitophrous vacuoles
